# APOBEC3 deletion increases the risk of breast cancer: a meta-analysis

**DOI:** 10.18632/oncotarget.11792

**Published:** 2016-09-01

**Authors:** Yali Han, Qichao Qi, Qin He, Meili Sun, Shuyun Wang, Guanzhou Zhou, Yuping Sun

**Affiliations:** ^1^ Department of Oncology, Jinan Central Hospital Affiliated to Shandong University, Jinan, 250013, China; ^2^ Department of Neurosurgery, Qilu Hospital of Shandong University and Brain Science Research Institute, Shandong University, Jinan, 250012, China; ^3^ Department of Endocrine and Metabolism, Qilu Hospital of Shandong University, Jinan, 250012, China; ^4^ Department of General Surgery, Qilu Hospital of Shandong University, Jinan, 250012, China

**Keywords:** APOBEC3, copy number variation, breast cancer, cancer susceptibility

## Abstract

Recently, a deletion in the human apolipoprotein B mRNA-editing enzyme catalytic polypeptide-like 3 (APOBEC3) gene cluster has been associated with a modest increased risk of breast cancer, but studies yielded inconsistent results. Therefore we performed a meta-analysis to derive a more precise conclusion. Six studies including 18241 subjects were identified by searching PubMed and Embase databases from inception to April 2016. Pooled odds ratios (ORs) and corresponding 95% confidence intervals (CIs) were evaluated under allele contrast, dominant, recessive, homozygous, and heterozygous models. All the analyses suggested a correlation of APOBEC3 deletion with increased breast cancer risk (D *vs* I: OR = 1.29, 95% CI = 1.23-1.36; D/D+I/D *vs* I/I: OR = 1.34, 95% CI = 1.26-1.43; D/D *vs* I/D+ I/I: OR = 1.51, 95% CI = 1.36-1.68; D/D *vs* I/I: OR = 1.75, 95% CI= 1.56-1.95; I/D *vs* I/I: OR = 1.28, 95% CI = 1.19-1.36). Stratified analysis by ethnicity showed that the relationship is stronger and more stable in Asians. In summary, our current work indicated that APOBEC3 copy number variations might have a good screening accuracy for breast cancer.

## INTRODUCTION

Breast cancer is the most frequently diagnosed cancer and the leading cause of cancer death among females worldwide [1]. Accumulating publications have reported that genetic factors play important roles in the pathogenesis of both sporadic and familial breast cancer [2–6]. Recent genome-wide association studies (GWASs) focusing on evaluating common single nucleotide polymorphisms (SNPs) have identified more than 70 genetic susceptibility loci for breast cancer [6–12]. However, these newly identified genetic factors, along with known high-penetrance breast cancer susceptibility genes, only explain a small portion of the heritability for this cancer [9].

The discovery of submicroscopic copy number variations (CNVs) present in our genomes has changed dramatically our perspective on DNA structural variation and disease. It is now thought that CNVs encompass more total nucleotides and arise more frequently than SNPs. CNVs may account for 13% of the human genome and have been supposed to explain some of the missing heritability for complex diseases after the findings from GWASs [13–16].

Recently a deletion in the APOBEC3 gene cluster was identified [17, 18]. This CNV is a deletion located between exon 5 of APOBEC3A and exon 8 of APOBEC3B, resulting in a fusion gene with a protein sequence identical to APOBEC3A, but with a 3’-UTR of APOBEC3B. It has been found that the deletion frequency was highly variable, rare in African and European populations (frequency of 0.9% and 6%, respectively), more common in East Asian and American populations (36.9% and 57.7%), and almost fixed in Oceanic populations (92.9%) [17]. Komatsu et al. first discovered an increased but statistically non-significant risk of breast cancer associated with APOBEC3 deletion in Japanese [19]. Then Long's study strongly indicated a positive correlation of APOBEC3 deletion with an elevated breast cancer risk in Chinese [20]. Later, Xuan's study in European and Rezaei's study in Iranian both showed the similar positive correlation with statistical power [21, 22]. However, the latest study by Göhler only revealed statistically non-significant results in Sweden and Marouf's study in Moroccans even indicated contrary results [23, 24]. As mentioned above, the results derived from current publications are inconclusive and even conflicting to each other, therefore we believe that it's necessary to perform a meta-analysis of available patient data to gain greater statistical power on this issue, with an expectation to obtain a pooled estimate much closer to the unknown truth and consequently to provide useful evidences and suggestions for early breast cancer screening and future investigation.

## RESULTS

### Study identification

For the process of eligible studies’ identification and selection, 16 publications were initially retrieved from PubMed and Embase databases. After further screening according to the inclusion and exclusion criteria, 6 relevant reviews and 4 molecular mechanism research studies were excluded. Finally, a total of 6 case-control studies with 18241 subjects were identified to be eligible for this meta-analysis [19–24]. The main characteristics of included studies were summarized in Table 1.

**Table 1:** Characteristics of studies on association between APOBEC3 gene deletion and breast cancer

Author	Year	Area	Ethnicity	Sample size		Source of Controls	Cases			Controls			Genotyping Method	*P* for HWE in controls	Quality Score
				Cases	Controls		I/I	I/D	D/D	I/I	I/D	D/D			
Marouf	2016	Morocco	Caucasian	226	200	PB	207	19	0	175	25	0	Real-time qualitative PCR (Taqman)	0.35	8
Göhler	2016	Sweden	Caucasian	782	1559	PB	633	142	5	1295	251	13	KASPar or Life Technologies assays	0.83	8
Rezaei	2015	Iran	Caucasian	262	217	PB	154	103	5	148	63	6	Real-time qualitative PCR	0.82	9
Xuan	2013	Europe	Caucasian	1671	1602	PB	1275	376	20	1279	314	9	Real-time qualitative PCR	0.03	8
Long	2013	China	Asian	5792	5830	PB	2045	2805	942	2530	2638	662	Real-time qualitative PCR	0.52	9
Komatsu	2008	Japan	Asian	50	50	PB	22	21	7	21	23	2	Real-time qualitative PCR	0.16	8

PB, population-based; PCR, polymerase chain reaction; HWE, Hardy-Weinberg equilibrium.

### Quantitative data analyses

Finally, 6 epidemiological individual studies including 8783 cases and 9458 controls were enrolled in this meta-analysis. All studies reported detailed number of three genotypes Insertion/Insertion (I/I), Insertion/Deletion (I/D), Deletion/Deletion (D/D). To be comprehensive, the correlation of APOBEC3 CNVs with breast cancer risk was analyzed by five different comparison models: allele contrast, dominant, recessive, homozygous, and heterozygous.

**Figure 1:**
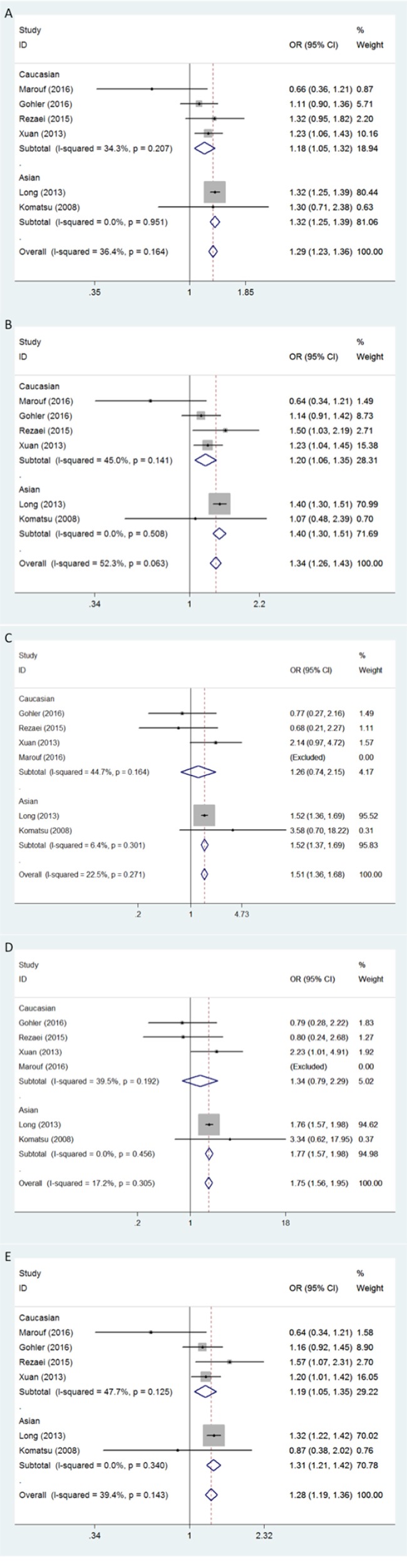
Forest plot of breast cancer risk associated with APOBEC3 gene deletion Models represented in **A.** allele contrast, **B.** dominant, **C.** recessive, **D.** homozygous, and **E.** heterozygous.

Firstly, in the allele contrast model, the APOBEC3 deletion variation was found to be significantly correlated with a higher breast cancer risk compared with the no-deletion allele (D *vs* I: OR = 1.29, 95% CI = 1.23-1.36) (Figure 1A). For the ethnicity-specific subgroup analysis, the results indicated that both Asians and Caucasians with the APOBEC3 deletion allele possessed an increased breast cancer susceptibility. Summary of the ORs and 95% CIs for breast cancer risk and APOBEC3 deletion under different genetic models was shown in Table 2.

**Table 2:** ORs and 95% CI for breast cancer risk and APOBEC3 deletion under different genetic models

Genetic models	n	OR [95% CI]	*P*_(OR)_	Model (method)	*I*-square (%)	*P*_(H)_	*P*_(Begg)_	*P*_(Egger)_
Allele contrast (D *vs* I)								
All	6	1.29 [1.23 - 1.36]	< 0.001	F (M-H)	36.4	0.164	0.260	0.115
Caucasian	4	1.18 [1.05 - 1.32]	0.005	F (M-H)	34.3	0.207	-	-
Asian	2	1.32 [1.25 - 1.39]	< 0.001	F (M-H)	0.0	0.951	-	-
Dominant model (D/D+I/D *vs* I/I)								
All	6	1.34 [1.26 - 1.43]	< 0.001	F (M-H)	52.3	0.063	0.452	0.119
Caucasian	4	1.20 [1.06 - 1.35]	0.004	F (M-H)	45.0	0.141	-	-
Asian	2	1.40 [1.30 - 1.51]	< 0.001	F (M-H)	0.0	0.508	-	-
Recessive model (D/D *vs* I/D+I/I)								
All	5	1.51 [1.36 - 1.68]	< 0.001	F (M-H)	22.5	0.271	1.000	0.809
Caucasian	3	1.26 [0.74 - 2.15]	0.389	F (M-H)	44.7	0.164	-	-
Asian	2	1.52 [1.37 - 1.69]	< 0.001	F (M-H)	6.4	0.301	-	-
Homozygous model (D/D *vs* I/I)								
All	5	1.75 [1.56 - 1.95]	< 0.001	F (M-H)	17.2	0.305	1.000	0.591
Caucasian	3	1.34 [0.79 - 2.29]	0.280	F (M-H)	39.5	0.192	-	-
Asian	2	1.77 [1.57 - 1.98]	< 0.001	F (M-H)	0.0	0.456	-	-
Heterozygous model (I/D *vs* I/I)								
All	6	1.28 [1.19 - 1.36]	< 0.001	F (M-H)	39.4	0.143	0.133	0.194
Caucasian	4	1.19 [1.05 - 1.35]	0.006	F (M-H)	47.7	0.125	-	-
Asian	2	1.31 [1.21 - 1.42]	< 0.001	F (M-H)	0.0	0.340	-	-

CI, confidence intervals; I, insertion; D, deletion; *P*_(OR)_, *P* for odds ratios; *P*_(H)_, *P* for heterogeneity; n, number of included studies; F, fixed-effect model; M-H, Mantel-Haenszel method.

The positive association between APOBEC3 deletion and breast cancer susceptibility was also found by the dominant model in both Asians and Caucasians (D/D+I/D *vs* I/I: for total, OR = 1.34, 95% CI = 1.26-1.43; for Asians, OR = 1.40, 95% CI = 1.30-1.51; for Caucasians, OR = 1.20, 95% CI = 1.06-1.35) (Figure 1B). Similarly, evidences from the heterozygous analysis also support a prominent association between APOBEC3 deletion and breast cancer risk, and no statistically significant ethnic variation was observed (I/D *vs* I/I: for total, OR = 1.28, 95% CI = 1.19-1.36; for Asians, OR = 1.31, 95% CI = 1.21-1.42; for Caucasians, OR = 1.19, 95% CI = 1.05-1.35) (Figure 1E).

As Marouf's study [24] included no individuals with D/D genotype, it was excluded in the comparison by recessive model (D/D *vs* I/D+I/I) and homozygous model (D/D *vs* I/I). After the exclusion, positive results were only found in Asians but not in Caucasians (D/D *vs* I/D+I/I: for total, OR = 1.51, 95% CI = 1.36-1.68,; for Asians, OR = 1.52, 95% CI = 1.37-1.69; for Caucasians: OR = 1.26, 95% CI = 0.74-2.15) (D/D *vs* I/I: OR = 1.75, 95% CI = 1.56-1.95; for Asians, OR = 1.77, 95% CI = 1.57-1.98; for Caucasians: OR = 1.34, 95% CI = 0.79-2.29) (Figure 1C and 1D).

### Sensitivity analysis

Sensitivity analysis was conducted by deleting one study at a time to examine the influence of individual study to the pooled ORs. No single study materially altered the pooled ORs when they were sequentially deleted, suggesting that our results were stable and robust (data not shown).

### Publication bias

**Figure 2:**
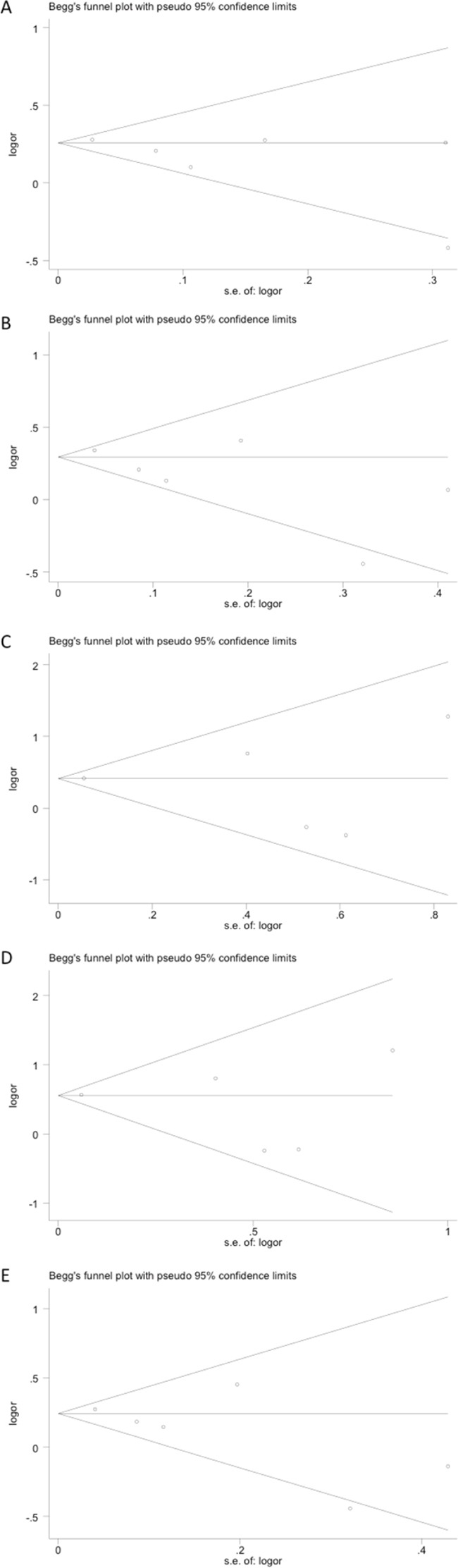
Publication bias tested by Begg's funnel plot Models represented in **A.** allele contrast, **B.** dominant, **C.** recessive, **D.** homozygous, and **E.** heterozygous.

The funnel plot revealed no obvious publication bias with a symmetrical distribution of study results around the pooled measurement of effect, indicating that publication bias was generally not a factor influencing the results (Figure 2). Egger's test and the Begg's test were employed to detect the potential publication bias, and also confirmed no statistically significant publication bias in this meta-analysis. All *P*-values from the Egger's test and the Begg's test were listed in Table 2.

## DISCUSSION

The APOBEC3 gene family, including APOBEC3A, APOBEC3B, APOBEC3C, APOBEC3D, APOBEC3E, APOBEC3F, APOBEC3G, and APOBEC3H, plays pivotal roles in intracellular defense against viral infections [25, 26]. The APOBEC3 genes family encodes cytosine deaminases that have been demonstrated to play key roles in innate immunity through their ability to mutagenize viral DNA and restrict viral replication [27, 28]. Recent advances in cancer genomics, together with biochemical characterization of the APOBEC3 enzymes, have implicated that APOBEC3 germline variations are specifically involved in many human cancers, including breast, bladder, lung (adenocarcinoma and squamous cell carcinoma), head/neck, and cervix [29, 30]. Moreover, the APOBEC3 gene may play a role in carcinogenesis by triggering DNA mutation [31]. Stephen Henderson and Tim Fenton have reviewed the evidences linking these enzymes to carcinogenesis including the potential mechanisms that misdirect APOBEC3 activity to the host genome, the links to viral infection, and the association between a common APOBEC3 polymorphism and cancer risk [27].

Up to now, emerging studies support a positive association between APOBEC3 CNVs and cancer risk [32, 33], particularly for breast cancer [19–24]. What's more, it's reported that the expression of APOBEC3 genes is regulated by estrogen (ER) [19], a hormone that plays a central role in the etiology of breast cancer. And Burns et al. provided evidences that APOBEC3B is overexpressed in breast tumors and cell lines and that the APOBEC3B mutation signature is statistically more prevalent in the breast tumor database of The Cancer Genome Atlas (TCGA) than is expected [34]. Also, APOBEC3B has recently been verified to be a marker of pure prognosis and poor outcomes for ER^+^ breast cancer, which strongly suggests that genetic aberrations induced by APOBEC3B contribute to breast cancer progression [35]. Additionally, a recent re-analysis of public breast cancer mutation data reported that breast cancers in carriers of the deletion show more mutations of the putative APOBEC-dependent genome-wide signatures than cancers in non-carriers. Their results suggested that the APOBEC3A/3B germline deletion allele confers cancer susceptibility through increased activity of APOBEC-dependent mutational processes, although the mechanism by which this occurs remains unknown [36].

Herein we performed a comprehensive databases search for all the eligible studies that reported the association between APOBEC3 CNVs and breast cancer risk. After pooling all the available data, we got a conclusion that the APOBEC3 deletion mutation significantly increases the risk of breast cancer, and the results are especially stable in Asians. OR (95% CI) of 1.29 (1.23-1.36) associated with deletion allele compared with no deletion allele strongly suggested a positive relationship between APOBEC3 deletion and breast cancer risk. And generally, consistent results were observed under the other four genetic models. When assessing by the homozygous and hetarozygous model, APOBEC3 deletion was also significantly associated with breast cancer risk, with OR (95% CI) of 1.28 (1.19-1.36) associated with one-copy deletion and 1.75 (1.56-1.95) associated with two-copy deletion compared with subjects with no deletion. The results present a possibility that the deletion copy number may be directly proportional to the breast cancer susceptibility.

In particular consideration of the influence of ethnicity, we conducted stratified analysis by ethnicity. Notably, the results showed that the relationship is weaker in Caucasians than in Asians. The ORs in Caucasian subgroup were always lower than those in Asian subgroup, and results in recessive model and homozygous model analysis revealed no statistical significance. Although analysis by other three genetic models showed positive results, we cannot convincingly conclude that APOBEC3 deletion would confer risk for breast cancer in Caucasian populations from the current assessment. More studies with large sample size in Caucasian populations are warranted to verify our results.

Our work has several strengths. Above all, this is by far the first meta-analysis evaluating the association between APOBEC3 CNVs and breast cancer risk. And it was comprehensively analyzed by five different comparison models. Besides, all the included studies were of high quality with NOS scores ranged from 8-9 score, which is critical for the reliability of our pooled results. What's more, no obvious heterogeneity was observed in all the analysis by five models and the ethnicity subgroup analysis further reduced the heterogeneity. Also, sensitivity analysis indicated no study to be deleted and no evident publication bias was detected. All these points significantly increased the statistical power of our analysis.

Despite its strengths, some limitations should be taken into consideration. Firstly, three of the included studies were based on a relatively small sample of less than 500 subjects and Long's study with 11622 subjects weights much higher than others. Secondly, only literatures in English were included in our study. Finally, many other factors could influence our analysis, such as distinct covariant factors, various genotyping methods among studies and non-coincident baseline characteristics of different samples. All of these potential discrepancies interfere with the standardization of our pooled data, but we were unable to conduct further stratified analyses for lack of detailed information. Thus, further studies investigating this issue should consider the factors mentioned above.

In summary, our current work indicates that a high copy number of APOBEC3 deletion confers risk for breast cancer, suggesting a possibility that APOBEC3 CNVs has a good screening accuracy for breast cancer.

## MATERIALS AND METHODS

### Search strategy

Potentially eligible articles were obtained through searching PubMed and Embase databases up to April 2016. The literature search was performed using free-text words combined with Medical Subject Headings (MeSH). Gene-specific terms (APOBEC3 or apolipoprotein B mRNA editing enzyme, catalytic polypeptide 3) were combined with polymorphism-specific terms (polymorphism or polymorphisms or variation or variations or variant or mutation or mutations or genotype or genotypes) and disease-specific terms (breast cancer or breast cancers) to retrieve eligible studies. References from retrieved articles were further screened manually for other potentially available reports.

### Inclusion and exclusion criteria

Inclusion criteria for studies were the following: (1) case-control or cohort study design; (2) evaluating associations between APOBEC3 gene deletion and breast cancer risk; (3) providing OR estimates with 95% CIs or sufficient data for calculation; (4) published in English; and (5) performed on humans. Exclusion criteria were the following: (1) reviews and comments; (2) insuffient data for calculation; (3) performed on animals; and (4) duplication of a previous publication.

### Study selection and data extraction

Data extraction was performed independently by two investigators (Y. Han and Q. Qi) and disagreements were adjudicated by a third reviewer (Q. He). For each study, general characteristics such as the first author, publication year, country and ethnicity of patients, sample size, and genotyping method were collected.

### Quality assessment

The Newcastle-Ottawa Scale and Agency for Healthcare Research and Quality (http://www.ohri.ca/programs/clinical_epidemiology/oxford.asp; maximum score = 9 points) was used to evaluate the methodological quality, which scored studies based on the selection of patients, the comparability of the groups, and the quality of the sampling process. A study awarded a score of 0-3, 4-6, or 7-9 was considered as a low-, moderate-, or high-quality study, respectively. Two authors (Y. Han and Q. Qi) independently assessed the study quality, and inconsistency was discussed with another reviewer-author (Q. He), who acted as an arbiter.

### Statistical analysis

To obtain a more comprehensive assessment of associations between APOBEC3 deletion and breast cancer risk, five different comparison models were used: allele contrast, dominant, recessive, homozygous and heterozygous. Risk estimates were expressed as ORs and 95% CIs. Heterogeneity arising from pooled individual studies was examined by the *I*^2^ test and Q test. Values of *P* > 0.10 for the Q test or *I*^2^ < 50% was considered lack of heterogeneity and a fixed-effect model was used; otherwise, a random-effect model was used. Subgroup analysis by ethnicity was used to detect and reduce potential source of heterogeneity among studies. Sensitivity analysis was conducted by excluding one study at a time. We depicted the Begg's funnel plot and computed the Egger regression asymmetry test to assess the probability of publication bias. Values of *P* < 0.05 were indicative of statistically significant publication bias. Data analyses were carried out using Stata software, version 11.0 (Stata Corporation; College Station, TX, USA). All *P* values were two sided and *P*-values < 0.05 were considered statistically significant.

## References

[1] Torre LA, Bray F, Siegel RL, Ferlay J, Lortet-Tieulent J, Jemal A (2015). Global cancer statistics, 2012. CA Cancer J Clin.

[2] Greenman C, Stephens P, Smith R, Dalgliesh GL, Hunter C, Bignell G, Davies H, Teague J, Butler A, Stevens C, Edkins S, O'Meara S, Vastrik I (2007). Patterns of somatic mutation in human cancer genomes. Nature.

[3] Hemminki K, Muller-Myhsok B, Lichtner P, Engel C, Chen B, Burwinkel B, Forsti A, Sutter C, Wappenschmidt B, Hellebrand H, Illig T, Arnold N, Niederacher D (2010). Low-risk variants FGFR2, TNRC9 and LSP1 in German familial breast cancer patients. Int J Cancer.

[4] Nik-Zainal S, Alexandrov LB, Wedge DC, Van Loo P, Greenman CD, Raine K, Jones D, Hinton J, Marshall J, Stebbings LA, Menzies A, Martin S, Leung K (2012). Mutational processes molding the genomes of 21 breast cancers. Cell.

[5] Peterlongo P, Catucci I, Colombo M, Caleca L, Mucaki E, Bogliolo M, Marin M, Damiola F, Bernard L, Pensotti V, Volorio S, Dall'Olio V, Meindl A (2015). FANCM c. 5791C>T nonsense mutation (rs144567652) induces exon skipping, affects DNA repair activity and is a familial breast cancer risk factor. Hum Mol Genet.

[6] Stephens PJ, Tarpey PS, Davies H, Van Loo P, Greenman C, Wedge DC, Nik-Zainal S, Martin S, Varela I, Bignell GR, Yates LR, Papaemmanuil E, Beare D (2012). The landscape of cancer genes and mutational processes in breast cancer. Nature.

[7] Cai Q, Zhang B, Sung H, Low SK, Kweon SS, Lu W, Shi J, Long J, Wen W, Choi JY, Noh DY, Shen CY, Matsuo K (2014). Genome-wide association analysis in East Asians identifies breast cancer susceptibility loci at 1q32.1, 5q14.3 and 15q26.1. Nat Genet.

[8] Couch FJ, Kuchenbaecker KB, Michailidou K, Mendoza-Fandino GA, Nord S, Lilyquist J, Olswold C, Hallberg E, Agata S, Ahsan H, Aittomaki K, Ambrosone C, Andrulis IL (2016). Identification of four novel susceptibility loci for oestrogen receptor negative breast cancer. Nat Commun.

[9] Fletcher O, Houlston RS (2010). Architecture of inherited susceptibility to common cancer. Nat Rev Cancer.

[10] Garcia-Closas M, Couch FJ, Lindstrom S, Michailidou K, Schmidt MK, Brook MN, Orr N, Rhie SK, Riboli E, Feigelson HS, Le Marchand L, Buring JE, Eccles D (2013). Genome-wide association studies identify four ER negative-specific breast cancer risk loci. Nat Genet.

[11] Ghoussaini M, Fletcher O, Michailidou K, Turnbull C, Schmidt MK, Dicks E, Dennis J, Wang Q, Humphreys MK, Luccarini C, Baynes C, Conroy D, Maranian M (2012). Genome-wide association analysis identifies three new breast cancer susceptibility loci. Nat Genet.

[12] Michailidou K, Hall P, Gonzalez-Neira A, Ghoussaini M, Dennis J, Milne RL, Schmidt MK, Chang-Claude J, Bojesen SE, Bolla MK, Wang Q, Dicks E, Lee A (2013). Large-scale genotyping identifies 41 new loci associated with breast cancer risk. Nat Genet.

[13] Eichler EE, Flint J, Gibson G, Kong A, Leal SM, Moore JH, Nadeau JH (2010). Missing heritability and strategies for finding the underlying causes of complex disease. Nat Rev Genet.

[14] Stankiewicz P, Lupski JR (2010). Structural variation in the human genome and its role in disease. Annu Rev Med.

[15] Tuzun E, Sharp AJ, Bailey JA, Kaul R, Morrison VA, Pertz LM, Haugen E, Hayden H, Albertson D, Pinkel D, Olson MV, Eichler EE (2005). Fine-scale structural variation of the human genome. Nat Genet.

[16] Willer CJ, Speliotes EK, Loos RJ, Li S, Lindgren CM, Heid IM, Berndt SI, Elliott AL, Jackson AU, Lamina C, Lettre G, Lim N, Lyon HN (2009). Six new loci associated with body mass index highlight a neuronal influence on body weight regulation. Nat Genet.

[17] Kidd JM, Newman TL, Tuzun E, Kaul R, Eichler EE (2007). Population stratification of a common APOBEC gene deletion polymorphism. PLoS Genet.

[18] McCarroll SA, Hadnott TN, Perry GH, Sabeti PC, Zody MC, Barrett JC, Dallaire S, Gabriel SB, Lee C, Daly MJ, Altshuler DM (2006). Common deletion polymorphisms in the human genome. Nat Genet.

[19] Komatsu A, Nagasaki K, Fujimori M, Amano J, Miki Y (2008). Identification of novel deletion polymorphisms in breast cancer. Int J Oncol.

[20] Long J, Delahanty RJ, Li G, Gao YT, Lu W, Cai Q, Xiang YB, Li C, Ji BT, Zheng Y, Ali S, Shu XO, Zheng W (2013). A common deletion in the APOBEC3 genes and breast cancer risk. J Natl Cancer Inst.

[21] Rezaei M, Hashemi M, Hashemi SM, Mashhadi MA, Taheri M (2015). APOBEC3 Deletion is Associated with Breast Cancer Risk in a Sample of Southeast Iranian Population. Int J Mol Cell Med.

[22] Xuan D, Li G, Cai Q, Deming-Halverson S, Shrubsole MJ, Shu XO, Kelley MC, Zheng W, Long J (2013). APOBEC3 deletion polymorphism is associated with breast cancer risk among women of European ancestry. Carcinogenesis.

[23] Gohler S, Da Silva Filho MI, Johansson R, Enquist-Olsson K, Henriksson R, Hemminki K, Lenner P, Forsti A (2016). Impact of functional germline variants and a deletion polymorphism in APOBEC3A and APOBEC3B on breast cancer risk and survival in a Swedish study population. J Cancer Res Clin Oncol.

[24] Marouf C, Gohler S, Filho MI, Hajji O, Hemminki K, Nadifi S, Forsti A (2016). Analysis of functional germline variants in APOBEC3 and driver genes on breast cancer risk in Moroccan study population. BMC Cancer.

[25] Stavrou S, Ross SR (2015). APOBEC3 Proteins in Viral Immunity. J Immunol.

[26] Wedekind JE, Dance GS, Sowden MP, Smith HC (2003). Messenger RNA editing in mammals: new members of the APOBEC family seeking roles in the family business. Trends Genet.

[27] Henderson S, Fenton T (2015). APOBEC3 genes: retroviral restriction factors to cancer drivers. Trends Mol Med.

[28] Warren CJ, Pyeon D (2015). APOBEC3 in papillomavirus restriction, evolution and cancer progression. Oncotarget.

[29] Burns MB, Temiz NA, Harris RS (2013). Evidence for APOBEC3B mutagenesis in multiple human cancers. Nat Genet.

[30] Roberts SA, Lawrence MS, Klimczak LJ, Grimm SA, Fargo D, Stojanov P, Kiezun A, Kryukov GV, Carter SL, Saksena G, Harris S, Shah RR, Resnick MA (2013). An APOBEC cytidine deaminase mutagenesis pattern is widespread in human cancers. Nat Genet.

[31] Suspene R, Aynaud MM, Guetard D, Henry M, Eckhoff G, Marchio A, Pineau P, Dejean A, Vartanian JP, Wain-Hobson S (2011). Somatic hypermutation of human mitochondrial and nuclear DNA by APOBEC3 cytidine deaminases, a pathway for DNA catabolism. Proc Natl Acad Sci U S A.

[32] Qi G, Xiong H, Zhou C (2014). APOBEC3 deletion polymorphism is associated with epithelial ovarian cancer risk among Chinese women. Tumour Biol.

[33] Zhang T, Cai J, Chang J, Yu D, Wu C, Yan T, Zhai K, Bi X, Zhao H, Xu J, Tan W, Qu C, Lin D (2013). Evidence of associations of APOBEC3B gene deletion with susceptibility to persistent HBV infection and hepatocellular carcinoma. Hum Mol Genet.

[34] Burns MB, Lackey L, Carpenter MA, Rathore A, Land AM, Leonard B, Refsland EW, Kotandeniya D, Tretyakova N, Nikas JB, Yee D, Temiz NA, Donohue DE (2013). APOBEC3B is an enzymatic source of mutation in breast cancer. Nature.

[35] Sieuwerts AM, Willis S, Burns MB, Look MP, Meijer-Van Gelder ME, Schlicker A, Heideman MR, Jacobs H, Wessels L, Leyland-Jones B, Gray KP, Foekens JA, Harris RS (2014). Elevated APOBEC3B correlates with poor outcomes for estrogenreceptor-positive breast cancers. Horm Cancer.

[36] Nik-Zainal S, Wedge DC, Alexandrov LB, Petljak M, Butler AP, Bolli N, Davies HR, Knappskog S, Martin S, Papaemmanuil E, Ramakrishna M, Shlien A, Simonic I (2014). Association of a germline copy number polymorphism of APOBEC3A and APOBEC3B with burden of putative APOBEC-dependent mutations in breast cancer. Nat Genet.

